# Glutathione Transferase Photoaffinity Labeling Displays GST Induction by Safeners and Pathogen Infection

**DOI:** 10.1093/pcp/pcad132

**Published:** 2023-10-31

**Authors:** Maria Font Farre, Daniel Brown, Maurice König, Brian J Killinger, Farnusch Kaschani, Markus Kaiser, Aaron T Wright, Jonathan Burton, Renier A L van der Hoorn

**Affiliations:** The Plant Chemetics Laboratory, Department of Biology, University of Oxford, Oxford OX1 3RB, UK; Chemistry Research Laboratory, Department of Chemistry, University of Oxford, Oxford, Oxfordshire OX1 3TA, UK; The Plant Chemetics Laboratory, Department of Biology, University of Oxford, Oxford OX1 3RB, UK; Voiland School of Chemical Engineering and Bioengineering, Washington State University, Pullman, WA 99164, USA; ZMB Chemical Biology, Faculty of Biology, University of Duisburg-Essen, Essen 45141, Germany; ZMB Chemical Biology, Faculty of Biology, University of Duisburg-Essen, Essen 45141, Germany; Voiland School of Chemical Engineering and Bioengineering, Washington State University, Pullman, WA 99164, USA; Department of Biology, Baylor University, Waco, TX 76798, USA; Department of Chemistry & Biochemistry, Baylor University, Waco, TX 76706, USA; Chemistry Research Laboratory, Department of Chemistry, University of Oxford, Oxford, Oxfordshire OX1 3TA, UK; The Plant Chemetics Laboratory, Department of Biology, University of Oxford, Oxford OX1 3RB, UK

**Keywords:** Agrochemical, Chemical proteomics, Glutathione transferase, GST, Immunity, Photoaffinity labeling, Safener

## Abstract

Glutathione transferases (GSTs) represent a large and diverse enzyme family involved in the detoxification of small molecules by glutathione conjugation in crops, weeds and model plants. In this study, we introduce an easy and quick assay for photoaffinity labeling of GSTs to study GSTs globally in various plant species. The small-molecule probe contains glutathione, a photoreactive group and a minitag for coupling to reporter tags via click chemistry. Under UV irradiation, this probe quickly and robustly labels GSTs in crude protein extracts of different plant species. Purification and mass spectrometry (MS) analysis of labeled proteins from *Arabidopsis* identified 10 enriched GSTs from the Phi(F) and Tau(U) classes. Photoaffinity labeling of GSTs demonstrated GST induction in wheat seedlings upon treatment with safeners and in *Arabidopsis* leaves upon infection with avirulent bacteria. Treatment of *Arabidopsis* with salicylic acid (SA) analog benzothiadiazole (BTH) induces GST labeling independent of NPR1, the master regulator of SA. Six Phi- and Tau-class GSTs that are induced upon BTH treatment were identified, and their labeling was confirmed upon transient overexpression. These data demonstrate that GST photoaffinity labeling is a useful approach to studying GST induction in crude extracts of different plant species upon different types of stress.

## Introduction

Glutathione transferases (GSTs) are a large and diverse family of enzymes that catalyze the conjugation of the tripeptide glutathione (γGlu–Cys–Gly) to a broad range of small-molecule substrates (Sylvestre-Gonon et al. 2019). GSTs have a conserved 3D structure with two conserved pockets: a glutathione-binding site (G-site) and a hydrophobic substrate–binding site (H-site) (Ding et al. 2017). The G-site is conserved among GSTs, stressing the importance of the correct binding and orientation of glutathione for their correct function. The H-site is a more variable pocket, responsible for the promiscuous binding of diverse substrates. Most GSTs are cytosolic and accumulate as soluble homodimers.

In plants, GSTs play important roles in biosynthetic pathways, agrochemical detoxification, plant defense and oxidative stress (Basantani and Srivastava 2007). Glutathione-conjugating GSTs of *Arabidopsis thaliana* include Tau (GSTU, 28 members), Phi (GSTF, 13 members), Theta (GSTT, three members), Lambda (GSTL, three members) and Zeta (GSTZ, two members) (Dixon et al. 2002, Dixon and Edwards 2010). GSTU, GSTF, GSTZ and GSTT classes contain a conserved Ser residue in their catalytic site and can also have peroxidase activity (Sylvestre-Gonon et al. 2019). By contrast, GSTLs are plant-specific Cys-GSTs that contain conserved catalytic Cys residues, and these GSTs reduce small molecules such as β-mercaptoethanol (βME) (Dixon and Edwards 2010). GSTU and GSTF classes are plant-specific and the most abundant and numerous GSTs in plants.

GSTs play a key role in the modification of exogenous compounds (xenobiotics) and their detoxification (Cummins et al. 2011). In agrochemical detoxification, GSTs are responsible for conjugating glutathione to herbicides, converting them into hydrophilic compounds with reduced mobility and toxicity. Metabolic herbicide resistance in weeds is associated with elevated levels of herbicide-catabolizing enzymes, including GSTs. In blackgrass (*Alopecurus myosuroides*) and ryegrass (*Lilium* spp.), the upregulation of GSTs is associated with resistance to multiple herbicides in resistant-field populations, with *Am*GSTF1 commonly upregulated in resistant blackgrass populations (Cummins et al. 2013, Tétard‐Jones et al. 2018, Dücker et al. 2019, 2020). Other examples are the high constitutive expression of GSTF2, associated with atrazine resistance in two waterhemp (*Amaranthus tuberculatus*) populations, and high GST levels in resistant populations of American sloughgrass (*Beckmannia syzigachne*) (Pan et al. 2016, Evans et al. 2017, Wang et al. 2020). Furthermore, safeners improve herbicide tolerance in crops by selectively inducing herbicide-metabolizing activities in cereal crops but not in weeds (Hatzios and Burgos, 2003). For instance, safener isoxadifen-ethyl reduces maize injury caused by the herbicide nicosulfuron associated with the induction of GSTs (Sun et al. 2017). Similarly, safeners fenclorim and metcamifen protect rice against injury by pretilachlor and clodinafop herbicides, respectively, again associated with GST induction (Taylor et al. 2013, Brazier-Hicks et al. 2020, Hu et al. 2020, 2021).

GSTs are also induced during biotic stress and play a role in plant defense (Nianiou-Obeidat et al. 2017, Gullner et al. 2018). Salicylic acid (SA) treatment induces the expression of Phi- and Tau-class GSTs in *Arabidopsis* and tomato (Sappl et al. 2004, Csiszár et al. 2014). A study on maize revealed a genetic linkage between a GST-encoding gene and multiple disease resistance to three different fungal leaf pathogens (Wisser et al. 2011). Likewise, silencing of *Nb*GSTF1 in *Nicotiana benthamiana* increased susceptibility to *Colletotrichum* infections (Dean et al. 2005).

The different roles of GSTs in plants are not yet fully understood, and this research field is hampered by the large number and diversity of GSTs. Here, we establish photoaffinity labeling of GSTs as an easy way to detect GSTs in various tissues of model plants, crops and weeds. We took advantage of a glutathione-based photoaffinity probe that successfully labeled recombinant human GSTs and endogenous GSTs from mouse liver and lung tissues (Stoddard et al. 2017). This photoaffinity probe contains glutathione, a benzophenone photoreactive group and an alkyne minitag that can be labeled with a fluorophore of biotin via click chemistry. We optimized labeling parameters, identified the probe targets and studied GSTs in plants responding to agrochemicals and biotic stress.

## Results

**Fig. 1:**
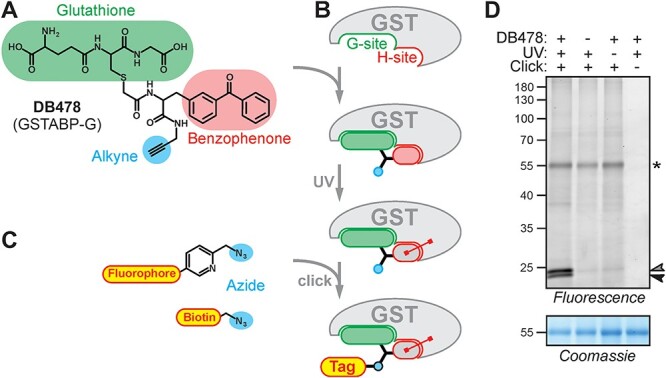
Photoaffinity labeling of GSTs. (A) The structure of the GST photoaffinity probe DB478, which was originally named GSTABP-G (Stoddard et al. 2017). (B) The procedure of photoaffinity labeling of GSTs. (C) Structures of the used reporters. (D) Photoaffinity labeling of *Arabidopsis* leaf extracts requires probe, UV light and click chemistry. An *Arabidopsis* leaf extract was incubated with and without a 5-μM probe and irradiated with UV light for 45 min and coupled to a fluorophore via click chemistry. Fluorescent proteins were visualized by in-gel fluorescence scanning. Coomassie staining is shown as loading control. Arrowheads: Specific labeling of GSTs; *aspecific labeling, probably from the large subunit of rubisco (RbcL).

To explore GST profiling in plants, we studied the chemical reactivity of the glutathione-based photoaffinity probe DB478 (**Fig. 1A**), which is a resynthesized replica of GSTABP-G (Stoddard et al. 2017). DB478 consists of three parts: (i) a glutathione moiety, which directs DB478 toward the glutathione-binding site (G-site) in GSTs; (ii) a benzophenone photoreactive group that facilitates the covalent cross-linking of the probe to a C-H bond in a nearby amino acid upon UV irradiation and (iii) an alkyne minitag for subsequent coupling to a fluorescent or affinity reporter tag via ‘click chemistry’ (**Fig. 1B**, **C**).

### Photoaffinity labeling of leaf extracts displays 23–24 kDa signals that depend on conditions

To study GST photoaffinity labeling, *Arabidopsis* leaf extracts were incubated with and without 5 μM DB478, irradiated with UV at 365 nm and coupled to a fluorophore with click chemistry. Fluorescence scanning of the labeled extracts separated on protein gels revealed two signals at 23–24 kDa, consistent with the molecular weight (MW) of GSTs (**Fig. 1D**). These 23–24 kDa signals are absent in the no-probe-control and require both UV treatment and click chemistry (**Fig. 1D**). Another, weaker signal at 55 kDa is caused by nonselective coupling of the reporter tag probably to rubisco as it does not require DB478 or UV exposure but depends on click chemistry (**Fig. 1D**).

**Fig. 2:**
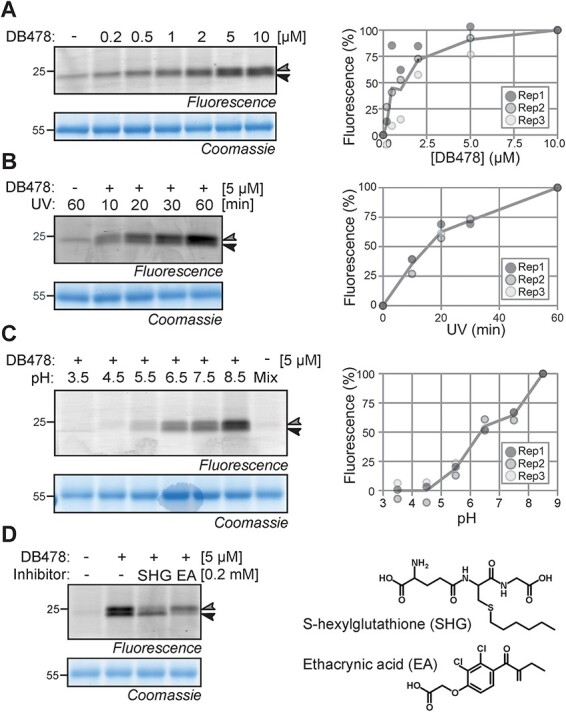
GST labeling depends on conditions. (A) Photoaffinity labeling saturates at 5-μM probe concentration. *Arabidopsis* leaf extracts were incubated with various probe concentrations and exposed to UV for 30 min. (B) Photoaffinity labeling requires >30 min UV treatment. *Arabidopsis* leaf extracts were incubated with a 5-μM probe and exposed to UV at various times. (C) Photoaffinity labeling requires pH > 6. (D) GST inhibitors differentially suppress photoaffinity labeling. *Arabidopsis* leaf extracts were incubated with a 5-μM probe at various pH and exposed to UV for 30 min. Samples were further analyzed as described in **Fig. 1D**. Plotted are signal intensities from three independent replicates normalized to the signal with the highest intensity.

We next characterized GST photoaffinity labeling further by studying the effect of probe concentration, UV exposure time, pH and presence of GST inhibitors. These experiments demonstrate that DB478 labeling reaches saturation at 5 μM (**Fig. 2A**) and that a 30-min UV exposure is sufficient to detect robust DB478 labeling (**Fig. 2B**). DB478 labeling is strongly dependent on pH, with poor labeling at acidic pH and increased labeling at neutral to basic pH (**Fig. 2C**). The two detected signals at 23–24 kDa are not differentially labeled at various probe concentrations, UV exposure times or pH. Therefore, the remaining experiments were performed with 5 μM DB478, at 45-min UV irradiation and at pH 7.5, which mimics the cytonuclear pH where GSTs reside.

We next confirmed GST labeling by competing DB478 labeling with known GST inhibitors. We tested S-hexylglutathione (SHG), a glutathione analog competing for the G-site of GSTs, and ethacrynic acid (EA), a substrate/inhibitor for the H-site of GSTs (Allocati et al. 2018). Interestingly, preincubation of *Arabidopsis* leaf extracts with SHG suppressed the labeling of the top signal, whereas preincubation with EA suppressed the labeling of the bottom signal (**Fig. 2D**), indicating that these signals are caused by different GSTs that have contrasting sensitivities to SHG and EA inhibitors.

### GSTs are the main targets of DB478

To identify the targets of DB478, *Arabidopsis* leaf extracts were incubated with 5 μM DB478, and labeled proteins were coupled to a biotin affinity tag for subsequent enrichment on streptavidin beads. Purified proteins were on-bead digested with Lys-C and trypsin, and digested peptides were identified by liquid chromatography–tandem mass spectrometry (LC-MS/MS). The pull-down assay was performed three times for DB478-labeled samples and three times for click control samples that followed the same procedure except no DB478 was added.

**Fig. 3:**
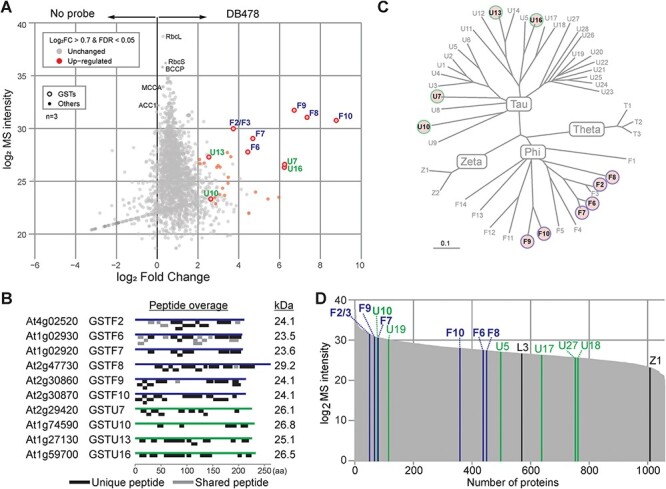
Proteomics confirms GST enrichment upon labeling. (A) GSTs are enriched in probe-labeled samples. *Arabidopsis* leaf extracts were labeled with and without a 5-μM probe, crosslinked and coupled to biotin via click chemistry and enriched on streptavidin beads. Beads were treated with trypsin, and released peptides were analyzed by MS. Plotted are the MS protein intensities plotted against the fold change for all proteins that were detected in all three replicates of the probe-labeled samples. Highlighted are all detected GST proteins (circles), abundant non-enriched proteins (RbcL, RbcS) and endogenously biotinylated proteins BCCP, MCCP and ACC1. FDR was calculated by the MaxLFQ algorithm of MaxQuant. (B) High protein coverage of GSTs. Unique and shared peptides identified by proteomics (A) were mapped onto the GST protein sequences. The predicted MW in kDa for each GST is on the left. (C) Identified GSTs highlighted in phylogeny of *Arabidopsis* GSTs. Tree is adapted from Wagner et al. (2002). Unrooted bootstrapped tree (*n* = 5000) is based on a multiple sequence alignment by ClustalX of the full-length protein sequences of all *Arabidopsis* GSTs. (D) In-solution digest (ISD) of the same *Arabidopsis* leaf extracts used for labeling. Shown are the proteins detected in all three replicates, ranked on their average LFQ intensity, with the detected GSTs indicated. GSTs that are enriched upon labeling are printed in bold.

After removing contaminants and retaining only proteins that were detected in all three samples of either the probe or the no-probe control, a total of 2,205 proteins were identified (**Supplementary Table S1**). Of the 29 proteins that were significantly enriched in the probe-labeled samples (**Supplementary Table S2**), we identified 10 GSTs from two different classes (Phi(F) and Tau(U); **Fig. 3A**). The highest enrichments (log2FC >6) and the highest mass spectrometry (MS) signal intensities are all caused by three members of the Phi class (GSTF8, GSTF9 and GSTF10) and two members of the Tau class (GSTU7 and GSTU16). We also detected GSTF2/3, GSTF6, GSTF7, GSTU10 and GSTU13 with high MS signal intensities but with lower but significant enrichments (log2FC >2). All 10 significantly enriched GSTs were detected with many unique peptides and 50–85% sequence coverage and have a predicted MW of 23.5–29.2 kDa (**Fig. 3B**), consistent with the fluorescent signals seen from protein gels. The other enriched proteins were detected at much lower MS intensities and do not have a predicted MW of 23–30 kDa. The majority of these other proteins were unchanged between the DB478 and click control (gray dots in **Fig. 3A**), and these include endogenously biotinylated proteins Biotin Carboxyl Carrier Protein (BCCP), Methylcrotonyl-CoA Carboxylase Alpha subunit (MCCA) and Acetyl-CoA Carboxylase-1 (ACC1) as well as abundant proteins such as the large and small subunits of rubisco (RbcL and RbcS, respectively). We consider them being caused by click chemistry background labeling, consistent with the background labeling seen on fluorescent gels (**Fig. 1D**).

Although we only identified GSTs from the Tau and Phi class, the identified that GSTs distribute over the phylogenetic tree of *Arabidopsis* GSTs (**Fig. 3C**; Wagner et al. 2002), indicating that DB478 can label diverse GSTs of these GST families. In-solution-digests of the proteomes used for labeling showed that all five Phi-class GSTs that were detected in the proteome were also detected upon labeling and enrichment (**Fig. 3D**, **Supplementary Table S3**). By contrast, except for GSTU10, five other members of the Tau class are present in the proteome but not enriched upon labeling (GSTU5, -U17, -U18, -U19 and -U27), indicating that not all GSTUs can be labeled by DB478. Conversely, we detected three Tau-class GSTs (GST-U7, -U13 and -U16) upon labeling that were not detected in the proteome, indicating their efficient enrichment upon labeling.

### GST photoaffinity labeling of other plant species

**Fig. 4:**
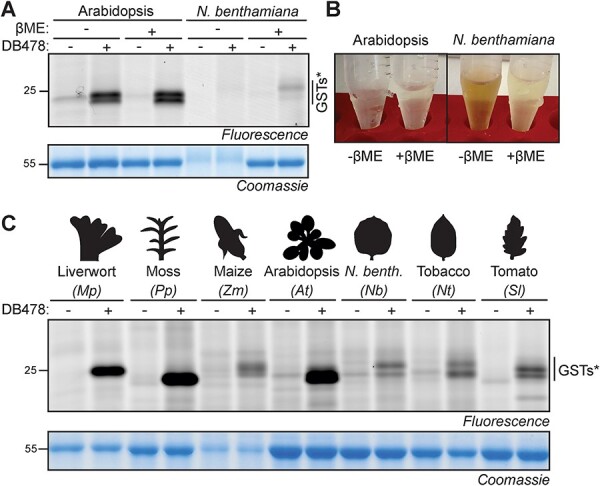
Putative GST labeling of other plant species. (A) Photoaffinity labeling of putative GSTs in *N. benthamiana* requires extraction with a reducing agent. Leaf extracts of *Arabidopsis* and *N. benthamiana* were generated with and without 3 mM βME, incubated with and without a 5-μM probe and exposed to UV light and visualized by fluorescence scanning and Coomassie staining. (B) βME avoids sample browning during labeling. (C) Putative GST labeling in leaf extracts of various plant species in the presence of βME. Leaf extracts were generated with 3 mM βME, incubated with and without a 5-μM probe and exposed to UV light and visualized by fluorescence scanning and Coomassie staining. *putative GST-derived signals.

To profile GSTs in other plant species, we tested leaf extracts of *N. benthamiana*, an important model plant used frequently for transient expression experiments (Bally et al. 2018). However, only weak signals were detected, in stark contrast to *Arabidopsis* leaf extracts (**Fig. 4A**). Leaf extracts of *N. benthamiana* usually turn brown quickly during labeling (**Fig. 4B**) in contrast to *Arabidopsis* leaf extracts. Browning is presumably caused by the oxidation of polyphenols by polyphenol oxidase (PPO) activity, which is frequently observed in plant extracts (Hamdan et al. 2022). Oxidation also results in cross-linking of proteins, which severely hampers their separation on protein gels, consistent with a strongly reduced RbcL signal from the Coomassie-stained gel (**Fig. 4A**). Protein oxidation can be prevented by performing the extraction in 5 mM βME, resulting in well-resolved RbcL signals on Coomassie-stained gels (**Fig. 4A**). Importantly, in the presence of βME, labeling of *N. benthamiana* leaf extracts with DB478 results in a specific 25 kDa signal, consistent with GST labeling (**Fig. 4A**).

We next tested if DB478 could label GSTs in additional plant species. We produced leaf extracts of two nonvascular plants (the liverwort *Marchantia polymorpha* and the moss *Physomitrium patens*) and five angiosperms (the monocot *Zea mays* and four eudicots *A. thaliana*, *N. benthamiana, Nicotiana tabacum* and *Solanum lycopersicum*). Labeling of leaf extracts with DB478 in the presence of βME consistently resulted in signals in the 25 kDa region, consistent with GST labeling (**Fig. 4C**). These signals vary in intensity and MW, reflecting the diversity of GSTs in various plant species.

### Safener treatment of wheat increases GST labeling

**Fig. 5:**
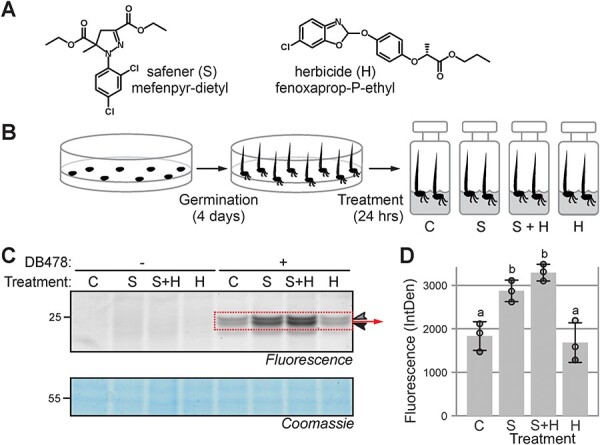
Safener induces GSTs in wheat. (A) Structures of safener and herbicide used in this experiment. (B) Experimental procedure of agrochemical treatment. (C) Safener treatment induces GST activities. Leaf extracts of seedlings incubated with/out safener and/or herbicide were labeled with DB478 and fluorescently labeled proteins were detected from protein gels by fluorescence scanning and quantified. Coomassie staining is shown as a loading control. (D) Quantified fluorescence of the 22–24 kDa signals from three biological replicates, analyzed by ANOVA. Groups a and b are statistically different from each other (*P* < 0.05). Error bars represent the standard deviation of three biological replicates.

GSTs are important detoxifying enzymes that contribute to herbicide resistance. Having established GST profiling on various plants, we investigated the effect of herbicides and safeners on GSTs. Fenoxaprop-P-ethyl is an extensively used post-emergence herbicide against grass weeds in wheat crops, usually applied in combination with safeners such as mefenpyr-diethyl (**Fig. 5A**, Taylor et al. 2013). To test whether the safener treatment induces GSTs, we incubated 4-d-old wheat seedlings in a liquid medium containing 100 µM safener (S), or herbicide (H), or both (S+H), or neither (C) for 1 d (**Fig. 5B**). Subsequent DB478 labeling revealed that seedlings treated with safener alone (S) or with safener and herbicide (S+H) have significantly elevated GST labeling, whereas seedlings treated with herbicide (H) have GST labeling that is similar to the control (C) (**Fig. 5C**). A possible synergistic effect of the dual S+H treatment was seen in various experiments but is not statistically significant (**Fig. 5C**). This experiment illustrates that DB478 labeling can be used to study the induction of GSTs upon agrochemical treatment, probably in both weeds and crops.

### Biotic stress and benzothiadiazole induce GSTs

**Fig. 6:**
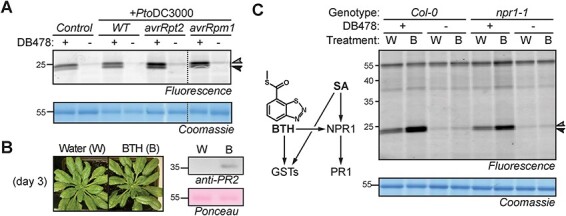
Pathogens and BTH induce GSTs independently of NPR1. (A) Avirulent *P. syringae* causes increased GST labeling in *Arabidopsis*. *Arabidopsis* leaves were infiltrated with *Pto*DC3000 wild-type (WT) or carrying plasmids encoding avrRpt2 or avrRpm1. Total extracts generated on day 2 were labeled with DB478, coupled to a fluorophore and analyzed by fluorescent scanning of protein gels. (B) BTH-treated plants. Adult *Arabidopsis* plants were watered with and without BTH for 3 d and proteins were extracted and analyzed by Western blot using anti-PR2 antibodies and Ponceau staining. (C) BTH-induced GST labeling is not dependent on NPR1. The samples of *Col-0* wild-type and *npr1-1* mutant plants treated with water/BTH for 3 d were labeled with 5 µM DB478, coupled to a fluorophore with click chemistry, followed by in-gel fluorescence scanning and Coomassie staining.

To study GST induction during biotic interactions, we infiltrated *Arabidopsis* leaves with wild-type *Pseudomonas syringae* pv. *tomato* DC3000 (*Pto*DC3000), which is pathogenic on *Arabidopsis*, and *Pto*DC3000 expressing avrRpt2 and avrRpm1, which are both avirulent on the *Arabidopsis Col-0* ecotype because this carries both *RPS2* and *RPM1* resistance genes, respectively. Interestingly, although wild-type *Pto*DC3000 had minimal impact on GST photoaffinity labeling, GST labeling is clearly elevated upon infection with both avirulent strains (**Fig. 6A**).

Since many responses triggered by avirulent bacteria are dependent on the stress hormone SA, we tested if treatment with benzothiadiazole (BTH), which is a mimic of SA, would be sufficient to induce GST labeling. BTH treatment for 3 d triggers Pathogenesis-Related (PR)2 protein accumulation without drastically reducing growth (**Fig. 6B**). GST photoaffinity labeling of these samples showed a robust and significant increase in GST labeling in BTH-treated samples when compared to the water-treated control samples (**Fig. 6C**).

Gene induction by BTH and avirulent pathogens involves the SA signaling pathway. Induction of many SA-responsive genes requires NPR1 (nonexpressor of PR1), which acts downstream of SA by activating TGACG-Binding (TGA) transcription factors that bind to *Activator sequence-1* (*as-1*) promoter elements of SA-responsive genes (Kumar et al. 2022). The *as-1* elements are also present in promotors of GST-encoding genes (Blanco et al. 2005). We therefore tested if BTH-induced GST labeling requires NPR1. Notably, increased GST labeling upon BTH treatment also occurs in the *npr1-1* mutant (**Fig. 6C**), demonstrating that NPR1 is not required for the increased GST labeling in samples from BTH-treated plants.

### BTH-induced GSTs do not increase immunity in transient assays

**Fig. 7:**
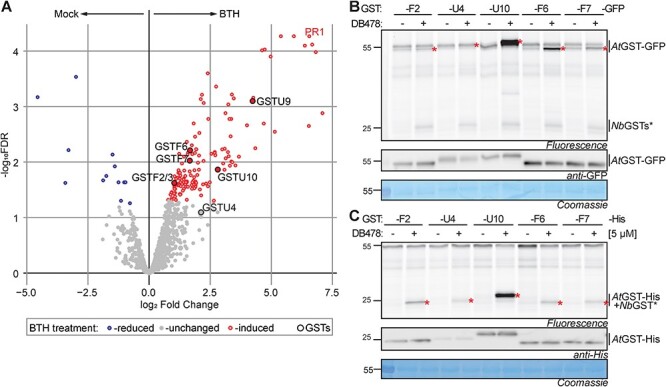
Transient overexpression of BTH-induced GSTs. (A) BTH-induced GSTs. Leaf extracts from water- and BTH-treated *Arabidopsis* plants were labeled with DB478 in triplicate. Labeled proteins were coupled to biotin-azide via click chemistry and enriched on streptavidin beads. On-bead digested proteins were analyzed by MS and plotted in volcano plots where log2 of fold change (log2FC) was plotted against their significance (-log_10_FDR) for three biological replicates. FDR was calculated by the MaxLFQ algorithm of MaxQuant. Proteins were significantly reduced (blue) induced (red) or unchanged (gray) upon BTH treatment. GSTs and PR1 are highlighted. (B) Labeling of GST–GFP fusion proteins upon agroinfiltration. *Arabidopsis* GSTs were fused to a C-terminal GFP tag and expressed in *N. benthamiana* by agroinfiltration. Extracts of agroinfiltrated leaves containing βME taken at 4 d upon agroinfiltration were labeled with and without 5 µM DB478, coupled to a fluorophore via click chemistry and visualized by in-gel fluorescence scanning. Fusions were detected by Western blot with anti-GFP antibody. Highlighted are labeled GFP-tagged GSTs (*) and endogenous GSTs (arrowheads). (C) Labeling of GST-His fusion proteins upon agroinfiltration. *Arabidopsis* GSTs were fused to a C-terminal His tag and expressed in *N. benthamiana* by agroinfiltration. Extracts of agroinfiltrated leaves containing βME taken at 4 d upon agroinfiltration were labeled with and without 5 µM DB478, coupled to a fluorophore via click chemistry and visualized by in-gel fluorescence scanning. Fusions were detected by Western blot with anti-GFP antibody. Highlighted are labeled GFP-tagged GSTs (*) and endogenous GSTs (arrowheads).

To investigate BTH-induced GSTs further, we labeled the total leaf extracts for *Arabidopsis* treated with water and BTH with DB478 and purified and identified labeled proteins by MS. Among the significantly BTH-induced proteins, we detected GSTU9 and U10 and GSTF2/3, F6 and F7, whereas GSTU4 just dropped below the significance level (**Fig. 7A**, **Supplementary Table S1**). Many additional proteins were detected at lower intensities, presumably caused by nonspecific binding. This includes PR1, which was detected as significantly induced upon BTH treatment (**Fig. 7A**), consistent with being a marker protein for SA signaling (Zhang et al. 1999).

To investigate the six BTH-induced GSTs further, we cloned them into agroinfiltration vectors with C-terminal His and GFP tags. While cloning of GSTU9 failed, the other five GSTs were successfully expressed by agroinfiltration of *N. benthamiana*, resulting in the detection of His-tagged and GFP-tagged fusion proteins (**Fig. 7B**, **C**). DB478 labeling of these samples showed clear labeling of all these GSTs, and strong labeling of GSTU10 and GSTF6 much beyond the level of endogenous GSTs, detected at 23–24 kDa (**Fig. 7B**, **C**).

## Discussion

In this study, we established photoaffinity labeling of GSTs in various plant species and used this to show safener-induced GST activities in wheat and biotic stress– induced and BTH-induced GSTs in *Arabidopsis*, independently from NPR1.

### Photoaffinity labeling of GSTs

Photoaffinity labeling is a robust way to study GSTs in plants. Conceptually, every glutathione-binding protein will be labeled with DB478. In reality, we only detected labeling of several GSTs from the Tau(U) and Phi(F) classes, consistent with the 23–24 kDa signals displayed on fluorescent protein gels. Labeling only Tau(U) and Phi(F) classes is not surprising because these are the two most abundant GST classes in plants (Cummins et al. 2011). Labeling of GFP/His-tagged GSTs upon overexpression by agroinfiltration confirms that these proteins are labeled with DB478.

A strong correlation between GST protein abundance and DB478 labeling indicates that DB478 is not selective to specific GSTs but will label diverse glutathione-conjugating GSTs in extracts. These observations are consistent with the detection of family-wide labeling of mammalian GSTs representing alpha, mu, pi, kappa, theta and zeta classes (Stoddard et al. 2017). Probe-enriched proteins are almost exclusively GSTs, demonstrating that this photoaffinity probe is highly selective. Although we detected several other proteins in pull-down assays using MS, these are also detected in the no-probe control and seem to be caused by nonselective click chemistry reactions, consistent with the higher background of the ‘click only’ control in fluorescent gels.

A similar set of GSTs has been identified with pull-down assays using glutathione beads (Sappl et al. 2004, Dixon and Edwards 2010), but these assays involve a longer purification protocol and require much more starting material. By contrast, photoaffinity labeling of GSTs can be performed on a 10 μg protein scale, without purification, following a simple, 3-h protocol, which allowed us to test and compare more conditions, treatments and mutants. We also demonstrated that GST photoaffinity labeling can be applied to a broad range of plant species, using examples of *Arabidopsis*, tomato, maize, wheat, liverwort and moss. This procedure can be applied to address a wide range of research questions, illustrated by our study of GST induction upon safener treatment in wheat and upon biotic stress in *Arabidopsis*.

Since this technique relies on photoaffinity labeling of the glutathione-binding site, we are monitoring a specific function of GSTs. And although labeling probably often reflects GST accumulation, we have seen deviations. GST inhibitors, for instance, can suppress labeling without affecting GST abundance (**Fig. 2D**). Also, the absence of labeling of endogenous GSTU19, despite being abundantly present (**Fig. 3**), might be the result of suppressed endogenous GST activity. In conclusion, photoaffinity labeling displays a functional layer of information on GSTs, which is distinct from the abundance displayed by transcriptomic and proteomic approaches. In most cases, however, the increased level of GST labeling seems to correlate with previously described increased transcriptional induction, e.g. upon safener or Bion treatments. This indicates that at these conditions, GSTs are not regulated by post-translational modifications or endogenous inhibitors. GST photoaffinity labeling is a robust tool to detect post-translational regulations of GSTs in the future, e.g. upon infection with plant pathogens.

### Safener induces GSTs in wheat

Using photoaffinity labeling of GSTs, we demonstrated that GSTs are induced in wheat seedlings upon treatment with safener mefenpyr-diethyl. Likewise, safener fenclorim transcriptionally induced five GSTs (GSTU25, -U24, -U19, -U4 and -U3) in *Arabidopsis* (Skipsey et al. 2011), and various safeners caused GSTU19 protein accumulation in *Arabidopsis* (DeRidder et al. 2002). Interestingly, cotreatment of safeners with herbicide seems to induce GST levels beyond that of safener alone. Although this possible synergism is not significant in our assays, this phenomenon is not much studied in plants. By contrast, the synergistic and antagonistic effects of simultaneous drug application on drug metabolizing enzymes have been extensively studied in mammals before (Tallarida 2001, Borisy et al. 2003, Roell et al. 2017). It is interesting to note that the molecular mechanism(s) underpinning the transcriptional induction of genes encoding metabolic enzymes by safeners and herbicides have not yet been resolved.

### Roles of GSTs in immunity

Using photoaffinity labeling and purification of the labeled proteins, we identified six BTH-induced GSTs from *Arabidopsis* leaves, which are largely consistent with SA-induced GST accumulation in *Arabidopsis* cell cultures (Sappl et al. 2004). GSTs might act in plant defense by detoxifying pathogen-derived phytotoxins and lipid hydroperoxides produced by the peroxidation of membranes (Gullner et al. 2018) or by catalyzing the biosynthesis of toxic metabolites, such as the role of GSTF6 in the production of the phytoalexin camalexin (Su et al. 2011). Redundancy within the GST family is often thought to hamper our understanding of the role of GSTs in immunity. Exceptions are that silencing of *NbGSTU1* in *N. benthamiana* increased susceptibility to the fungal pathogen *Colletotrichum destructivum* (Dean et al. 2005), and the *gstf9* mutant of *Arabidopsis* was more susceptible to the fungus *Verticilium dahliae* (Gong et al. 2018). By contrast, GST overexpression can overcome the redundancy problem with examples being the overexpression of cotton *Ga*GSTF9 in *Arabidopsis* increasing resistance to Verticillium wilt (Gong et al. 2018) and overexpression of *Lr*GSTU5 of royal lilly in tobacco increasing resistance to *Fusarium oxysporum* (Han et al. 2016).

### BTH-induced GST labeling is independent of NPR1

Our observation that avirulent bacteria and BTH induce GST labeling in *Arabidopsis* is consistent with the observation that SA induces the accumulation of GST proteins in *Arabidopsis* cell cultures, which correlates with their transcriptional induction (Sappl et al. 2004). That BTH-induced GST labeling also occurs in the *npr1* mutant is counterintuitive because BTH induces PR genes via NPR1. However, our observation that NPR1 is dispensable for BTH-induced GST induction is consistent with the literature. NPR1 directly binds TGA transcription factors that bind *as-1/ocs* elements in promoters of late SA-responsive genes (Kumar et al. 2022). Also, many GST-encoding genes, including *GSTF8, GSTU7* and *GSTU19*, have *as-1/ocs* elements in their promoter that are essential for induction by SA (Zhang and Singh 1994, Chen and Singh 1999, Blanco et al. 2005). But GSTs are early SA-responsive genes and these are also induced without NPR1. For instance, SA-induced expression of *GSTF9 (GST6)* and *GSTU7 (GST25)* does still occur in the *npr1* mutant even though SA-induced expression of *PR1* is absent (Uquillas et al. 2004, Blanco et al. 2005). Likewise, transcriptional induction of the promoter of maize *Zm*GSTL1 by safeners in transgenic *Arabidopsis* was SA dependent and was blocked in the quadrupole *tga2/3/5/6* mutant but unaltered in the *npr1* mutant (Behringer et al. 2011). Collectively, these data indicate that although induction of late SA-responsive genes is dependent on NPR1, early SA-responsive genes such as GSTs are induced via TGA transcription factors independently of NPR1. Studies on the induction of GST-encoding *GNT35* in tobacco has indicated a role for oxidative signaling because *GNT35* induction by SA can be blocked with antioxidants, and oxidative stress can induce GST expression in the absence of SA (Garretón et al. 2002).

Further elucidation of the GST induction pathway will increase our understanding of how safeners induce herbicide resistance and how pathogens induce the detoxification machinery. Photoaffinity labeling of GSTs can be a helpful new instrument in further investigations since it reports on global GST induction and can be used on any plant species upon treatments with agrochemicals and pathogens.

## Conclusion

We have established a simple and robust assay to detect GSTs with photoaffinity labeling in extracts of various plant species and shown that this method can be used to detect GST induction upon agrochemical treatment and pathogen infection. We have used proteomics to show the labeling of several Tau- and Phi-class GSTs and identified BTH-induced GSTs, which we confirmed by labeling transiently expressed tagged GSTs. We confirm that GST induction by SA analog BTH is independent of NPR1 and show that overexpression of defense-related GSTs does not affect immunity in transient disease assays.

## Materials and Methods

### Chemical probes, inhibitors and agrochemicals

GST inhibitors and agrochemicals were purchased from Sigma-Aldrich: EA (Sigma-Aldrich, St. Luis, MA, SML1083), SHG (Sigma-Aldrich, H6886), mefenpyr-diethyl (Sigma-Aldrich, 46302) and fenoxaprop-P-ethyl (Sigma-Aldrich, 36851). DB478 was resynthesized as described before (Stoddard et al. 2017), with minor modifications (see **Supplementary Methods**). Briefly, (tert-butoxycarbonyl)-L-phenylalanine was subjected to pentaflurophenyl trifluoroacetate to generate the respective pentafluorophenyl ester *in situ*, which was reacted with propargyl amine to give amide DB475 in 83% yield. The Boc-protecting group was removed, and the free amine of the resulting DB476 was acylated with bromoacetyl bromide in the presence of excess triethylamine to produce the α-bromoacetamide DB477 in 54% yield over both steps combined. The reaction of DB477 with reduced glutathione gave DB478 in 99% yield. Aliquots of DB478 are available upon request.

### Plant materials, growth conditions and agrochemical treatments

*Arabidopsis* (*A. thaliana*) ecotype Columbia plants were grown on soil at 25°C at short-day conditions (8/16-h light/dark regime) in a growth chamber. *Nicotiana benthamiana*, maize (*Z. mays*), tobacco (*N. tabacum*) and tomato (*S. lycopersicum*) were grown in soil at standard greenhouse conditions. Liverwort (*M. polymorpha*) and moss (*Physcomitrium patens*) were grown under sterile conditions as described by Thamm et al. (2020) and Moody et al. (2021), respectively. For the BTH treatment, 5-week-old *Arabidopsis* plants were grown on soil in a controlled environment (short-day conditions, 25°C). BION granules (Syngenta, Jealott’s Hill, UK) were dissolved in water to a final concentration of 1 μM BTH, and *Arabidopsis* plants were watered with or without BTH over three consecutive days. *Arabidopsis* leaf tissue was harvested on day 4 and frozen in liquid nitrogen till further use. Winter wheat (*Triticum aestivum*) seeds were sterilized with 30% ethanol for 5 min, 30% bleach for 10 min and rinsed six times with sterile water. Sterilized seeds were plated in Murashige and Skoog agar plates (half-strength Murashige and Skoog basal [Duchefa-Biochemie, M0222] salt mixture containing 0.8% agar) and stratified at 4°C for 1 d. For germination, seeds were placed at 25°C under long-day conditions (16/8-h light/dark regime). After 4 d, germinated wheat seedlings were transferred into Hoagland’s liquid medium (half-strength Hoagland’s No.2 Basal Salt Mixture [Sigma-Aldrich, H2395]). After 1-d adaptation, seedlings were treated with 100 μM mefenpyr-diethyl, fenoxaprop-P-ethyl, mefenpyr-diethyl or fenoxaprop-P-ethyl or an equal amount of dimethyl sulfoxide (DMSO) solvent. Seedling tissue was harvested 24 h after treatment and frozen in liquid nitrogen until used.

### Cloning

Used and generated plasmids are summarized in **Supplementary Table S4**. The primers used for sequence amplification are summarized in **Supplementary Table S5**. Golden Gate Modular Cloning Kit (Weber et al. 2011) and Golden Gate Modular Cloning Toolbox for plants (Engler et al. 2014) were used for cloning. The binary vector pJK268c containing the P19 silencing inhibitor (pL1V2-P19-F2) was used as a binary vector (Kourelis et al. 2020). The sequences encoding *Arabidopsis* GSTs (GSTF2, GSTU4, GSTU10, GSTF6 and GSTF7) were amplified from *Arabidopsis* complementary DNA (cDNA) using the forward and reverse primer pairs summarized in **Supplementary Table S5**. Using BsaI restriction sites, PCR products were combined with GG1-55, GG1-57, GG1-70 or GG1-78 (**Supplementary Table S4**) to assemble the corresponding expression constructs. Using BpiI restriction sites, PCR products were first cloned into the level-0 module GG1-01 and later transferred to pJK268c together with GG1-55, GG1-57, GG1-70 or GG1-78 in a BsaI ligation reaction. Generated expression constructs were transformed into *Escherichia coli* for their amplification and sequencing. Afterward, constructs were transformed into *Agrobacterium* (*Agrobacterium tumefaciens*) strain GV3101, carrying the pMP90 helper plasmid. *Agrobacterium* transformants were selected on lysogeny broth (LB) agar plates (10 g/l NaCl, 10 g/l tryptone, 5 g/l yeast extract and 15 g/l agar) containing 50 μM gentamycin, 25 μM rifampicin and 50 μM kanamycin. Single colonies were picked and grown in liquid LB (10 g/l NaCl, 10 g/l tryptone and 5 g/l yeast extract) containing the same antibiotic used before. Glycerol stocks of the selected transformants were prepared by mixing *Agrobacterium* cultures with 50% sterile glycerol in a 1:1 ratio.

### Agroinfiltrations

*Nicotiana benthamiana* plants were grown on soil at greenhouse conditions described before. *Agrobacterium tumefaciens* GV3101-pMP90 carrying the desired expression constructs were grown overnight (approximately 18 h) at 28°C with agitation in LB media containing 50 μM gentamycin, 25 μM rifampicin and 50 μM kanamycin. Bacterial cells were collected by centrifugation at 1,000×*g* for 10 min at room temperature, washed with infiltration buffer (10 mM MES, 10 mM MgCl_2_, pH 5.6 and 150 μM acetosyringone), diluted to an optical density at 600 nm (OD600) of 0.5. Bacteria were incubated at 28°C for 2 h, and afterward, the two new fully expanded leaves of 4- to 5-week-old *N. benthamiana* plants were hand infiltrated with the bacterial suspensions with the help of a needle-less 10 ml syringe. Agroinfiltrated *N. benthamiana* leaf tissue was collected at 4 d post-infiltration (dpi) or used for infection assays. For *P. infestans* infection assays, *N. benthamiana* leaves were half infiltrated with the empty vector (EV) control and half infiltrated with the construct of interest (GST-expressing construct).

### Protein extraction

Leaf tissue from various plant species was frozen in liquid nitrogen and ground to fine powder with a mortar and pestle. *Arabidopsis* total proteins were extracted in cold homogenization buffer (100 mM Tris-HCl pH 7.4) in a 1:3 weight-to-volume ratio. For the other plant species, total proteins were extracted in cold Tris-HCl buffer containing 3 mM βME. The extracts were incubated for 20 min rotating at 4°C for complete homogenization and centrifuged at 5,000×*g* for 20 min at 4°C. Supernatants containing soluble proteins were filtered through a layer of miracloth filter and centrifuged again at 5,000×*g* for 20 min at 4°C. Frozen wheat seedlings were ground to fine powder with the help of a tissue lyser (QIAGEN, Hilden, Germany, TissueLyser II) in 1.5 ml safe-lock Eppendorf tubs containing three 2.4-mm metal beads. Next, total proteins were extracted in cold Tris-HCl homogenization buffer in a 1:3 weight-to-volume ratio and cleared by centrifugation at 10,000×*g* for 10 min at 4°C. For all extractions, total protein concentration was determined by a DC protein assay Kit (Bio-Rad, Hercules, CA, 5000116) with bovine serum albumin (BSA) standards. Protein concentration was adjusted to 0.5 mg/ml for each sample, and the resulting samples were used for labeling without freeze-thawing.

### Labeling and click chemistry

DB478 was prepared at 50–1,000 μM stock solutions in DMSO. Samples of 50 μl were incubated with or without DB478 at the indicated probe concentration (usually 5 μM) and left 20 min incubating before UV radiation (365 nm, in microtiter plates on ice) for the indicated amount of time (usually 45 min). Equal volumes of DMSO were added to no-probe controls. For inhibition experiments, 50 μl extracts were preincubated with competitor molecules, 0.2 mM of SHG or EA, for 15 min before DB478 labeling. Labeling reactions were stopped by adding 1:4 (v/v) cold acetone and precipitated by 3 min centrifugation at maximum speed using a benchtop centrifuge. Protein pellets were resuspended in 44.5 μl of phosphate-buffered saline–sodium dodecyl sulfate (PBS-SDS) buffer (PBS pH 7.4 with 1% (w/v) SDS) and heated for 3 min at 90°C. For click chemistry, 44.5 μl of labeled proteins were incubated with 2 μM Cy5 Picolyl Azide (VectorLabs, Newarc, CA, 50 μM stock in DMSO) or Fluorescein Picolyl Azide (Click Chemistry Tools, 50 μM stock in DMSO) and a premixture of 100 μM tris((1-benzyl-1H-1,2,3-triazol-4-yl) methyl)amine (TBTA; 3.4 mM stock in DMSO:t-butanol 1:4), 2 mM tris(2-carboxyethyl)phosphine hydrochloride (TCEP; 100 mM stock in water) and 1 mM copper(II) sulfate (CuSO_4_; 50 mM stock in water). Samples were incubated for 1 h at room temperature in the dark and quenched by acetone precipitation. Protein pellets were resuspended in 50 μl of 2× gel loading buffer (100 mM Tris-HCl pH 6.8, 200 mM dithiotreitol, 4% SDS, 20% glycerol and 0.02% bromophenol blue) and heated for 5 min at 90°C.

### In-gel fluorescence scanning and Western blot

Labeled proteins were loaded and separated on 10–12% SDS-PAGE acrylamide gels at 150 V. Fluorescence scanning of acrylamide gels was performed on an Amersham Typhoon 5 scanner (GE Healthcare Life Sciences, Chalfont St Giles, UK) using excitation and emission wavelengths of ex488/em520 and ex635/em670 for Cy2 and Cy5 scanning, respectively. Scanned gels were stained with Coomassie Brilliant Blue G-250. For Western blot, proteins separated in SDS-PAGE gels were transferred to a PVDF membrane using Trans-Blot Turbo Transfer System (Bio-Rad). For anti-His and anti-GFP blots, membranes were blocked in 5% (w/v) milk in TBS-T (50 mM Tris-HCl pH 7.6, 150 mM NaCl and 0.1% Tween-20) overnight at 4°C and incubated in 1:5,000 anti-GFP-HRP (Abcam, Cambridge, UK, ab6663) or anti-6xHis-HRP (Invitrogen, #R931-25) in TBS-T/milk 5% for 1.5 h at room temperature. For anti-PR2 blots, membranes were blocked in TBS-T/milk 5% overnight at 4°C and incubated in 1:2,000 anti-PR2 (Agrisera, Vannas, Sweden, AS122366) in TBS-T/milk 5% for 1.5 h at room temperature. Membranes were washed three times for 5 min in TBS-T at room temperature and incubated in 1:10,000 secondary anti-Rabbit-HRP (Invitrogen, #31460) in TBS-T/Milk 5% for 1 h at room temperature. Before scanning, all blots were washed six times for 5 min in TBS-T/PBS-T and a last wash for 5 min in TBS/PBS. Clarity Western ECL substrate (Bio-Rad) was used for chemiluminescent protein detection.

### Large-scale labeling and affinity purification

A volume of 1 ml protein extracts (0.5 mg/ml protein concentration) was incubated with or without 5 μM DB478 as described in the ‘Labeling and click chemistry’ section. Labeling reactions were stopped by methanol/chloroform precipitation (addition of 4 volumes of ice-cold methanol, 1 volume of ice-cold chloroform and 3 volumes of water) and precipitated by 40 min centrifugation at 4,000×*g* at 4°C. Protein pellets were resuspended in 1 ml of PBS-SDS buffer by bath sonication and heated at 90°C for 5 min. Labeled proteins were biotinylated with 20 μM azide-PEG3-biotin (Sigma-Aldrich, 2 mM stock in DMSO) using click chemistry as described in the ‘Labeling and click chemistry’ section. Samples were incubated for 1 h at room temperature in the dark, quenched by the addition of 10 mM ethylenediaminetetraacetic acid (EDTA) and precipitated via the methanol/chloroform method. Protein pellets were resuspended in 1.5 ml of 1.2% (w/v) SDS dissolved in PBS by bath sonication and diluted by adding 7.5 ml of PBS. The resulting solution was incubated with 100 μl of pre-equilibrated Pierce High Capacity Streptavidin Agarose beads (Thermo Fisher Scientific, Waltham, MA, 10302384) for 2 h at room temperature. Agarose beads containing the labeled proteins were collected by centrifugation at 1,000×*g* for 5 min at room temperature. Agarose beads were washed three times with 10 ml of PBS–1% SDS buffer, three times with PBS and a final wash with 10 ml of water.

### On-bead trypsin and Lys-C digestion

For on-bead digestion, the captured agarose beads containing the labeled proteins were treated with 500 μl 6 M urea dissolved in PBS and 10 mM TCEP (200 mM stock in water) for 15 min at 65°C. A final concentration of 20 mM 2-chloroacetamide (CA, 400 mM stock in water) was added, and the sample was incubated for 30 min at 35°C in the dark. The reaction was diluted by the addition of 950 μl of PBS, and the supernatant was removed by centrifuging the beads at 500 g for 2 min. 5 μg of Lys-C (hermo Fisher Scientific) was reconstituted in 100 μl 1 M urea/50 mM Tris-HCl buffer at pH 8 and was added to the beads and left incubating at 37°C for 3 h. Trypsin digestion solution of 100 μl (Promega, Madison, WI, 5 μl of reconstituted trypsin was dissolved in 100 μl 50 mM Tris-HCl buffer at pH 8) was added to the beads and left incubating at 37°C overnight. The supernatant containing the digested peptides was dried by vacuum centrifugation and submitted for MS analysis.

### Sample preparation for MS

The cleared tryptic digests were then desalted on homemade C18 StageTips as described by Rappsilber et al. (2007). Briefly, peptides were immobilized and washed on a two-disk C18 StageTip. After elution from the StageTips, samples were dried using a vacuum concentrator (Eppendorf, Hamburg, Germany), and the peptides were taken up in 0.1% formic acid solution (15 μl) and directly used for LC-MS/MS experiments (see later for details).

### LC-MS/MS settings

MS experiments were performed on an Orbitrap LUMOS instrument (Thermo Fisher Scientific) coupled to an EASY-nLC 1200 ultra-performance liquid chromatography (UPLC) system (Thermo Fisher Scientific). The UPLC was operated in the one-column mode. The analytical column was a fused silica capillary (75 µm × 46 cm) with an integrated fritted emitter (New Objectives PF360-75-15-N-5) packed in-house with 1.9 µm Reprosil-Pur 120 C18-AQ (Dr. Maisch). The analytical column was encased by a column oven (Sonation PRSO-V2) and attached to a nanospray flex ion source (Thermo Fisher Scientific). The column oven temperature was set to 50°C during sample loading and data acquisition. The LC was equipped with two mobile phases: solvent A (0.1% formic acid (FA), 99.9% H_2_O) and solvent B [0.1% FA, 80% acetonitrile (ACN) and 19.9% H_2_O]. All solvents were of UPLC grade (Honeywell, Charlotte, NC). Peptides were directly loaded onto the analytical column with a maximum flow rate that would not exceed the set pressure limit of 980 bar (usually around 0.4–0.6 µl/min). Peptides were subsequently separated on the analytical column by running a 120-min gradient of solvent A and solvent B (start with 5% B; gradient 5–44% B for 90:00 min; gradient 44–100% B for 20:00 min; 100% B for 10 min) at a flow rate of 300 nl/min. The mass spectrometer was controlled by the Orbitrap Fusion Lumos Tune Application (version 3.3.2782.28) and operated using the Xcalibur software (version 4.3.73.11). The mass spectrometer was set in the positive ion mode. The ionization potential (spray voltage) was set to 2.2 kV. Source fragmentation was turned off. Precursor ion scanning was performed in the Orbitrap analyzer (FT; Fourier transform mass spectrometer) in the scan range of m/z 375-1750 and at a resolution of 240,000 with the internal lock mass option turned on (lock mass was 445.120025 m/z, polysiloxane) (Olsen et al. 2005). Automatic gain control (AGC) was set to ‘standard’ and acquisition time to ‘50 ms’. Product ion spectra were recorded in a data-dependent fashion in the Iontrap (IT) at a rapid scan rate. Peptides were analyzed using a ‘top speed’ regime (repeating cycle of full precursor ion scan (AGC target ‘standard’ and acquisition time ‘auto’) followed by dependent MS2 scans for 3 s (minimum intensity threshold 3 × 10^3^). The MS2 precursor ions were isolated using the quadrupole (isolation window 1.2 m/z), and fragmentation was achieved by higher-energy C-trap dissociation (HCD) (normalized collision energy set to 35%). During MS2 data acquisition, dynamic ion exclusion was set to 40 s. Only charge states between 2 and 7 were considered for fragmentation.

### Peptide and protein identification using MaxQuant

RAW spectra were submitted to an Andromeda (Cox et al. 2011) search in MaxQuant (v1.6.10.43) using the default settings (Cox and Mann 2008). Label-free quantification and match-between-runs were activated (Cox et al. 2014). The MS/MS spectra data were searched against the Uniprot *A. thaliana* reference database (UP000006548_3702.fasta, 39,350 entries, downloaded 19 November 2020). All searches included a contaminant database search (as implemented in MaxQuant, 245 entries). The contaminant database contains known MS contaminants and was included to estimate the level of contamination. Andromeda searches allowed oxidation of methionine residues (16 Da) and acetylation of the protein N-terminus (42 Da) as dynamic modifications and the static modification of cysteine (57 Da, alkylation with iodoacetamide). Enzyme specificity was set to ‘Trypsin/P’ with two missed cleavages allowed. The instrument type in Andromeda searches was set to Orbitrap, and the precursor mass tolerance was set to ±20 ppm (first search) and ±4.5 ppm (main search). The MS/MS match tolerance was set to ±0.5 Da. The peptide spectrum matches False Discovery Rate (FDR) and the protein FDR was set to 0.01 (based on the target-decoy approach). The minimum peptide length was seven amino acids. For protein quantification, unique and razor peptides were allowed. Modified peptides were allowed for quantification. The minimum score for modified peptides was 40. Label-free protein quantification was switched on, and unique and razor peptides were considered for quantification with a minimum ratio count of 2. Retention times were recalibrated based on the built-in nonlinear time-rescaling algorithm. MS/MS identifications were transferred between LC-MS/MS runs with the ‘match-between-runs’ option in which the maximal match time window was set to 0.7 min and the alignment time window set to 20 min. The quantification is based on the ‘value at maximum’ of the extracted ion current. At least two quantitation events were required for a quantifiable protein. Further analysis and filtering of the results was done in Perseus v1.5.5.3. (Tyanova et al. 2016). For quantification, we combined related biological replicates to categorical groups and investigated only those proteins that were found in at least one categorical group in a minimum of four out of five biological replicates. Comparison of protein group quantities (relative quantification) between different MS runs is based solely on the Label Free Quantifications (LFQs) as calculated by MaxQuant, MaxLFQ algorithm (Cox et al. 2014).

Filtering of results was done in Perseus version 1.6.13.0 (Tyanova et al. 2016). Briefly, data were filtered against contaminants, and only those proteins found in at least one group in all three replicates were considered. Further analysis and graphs were performed using the Qfeatures and ggplot R packages (Gatto 2021). Imputed values were generated using a missing not at random (MNAR) and missing at random (MAR) mixed imputation over the whole matrix, and the fold change and adjusted *P*-values (adj. *P* Val) were calculated using the FDR method over the three biological replicates. The correct clustering of the biological replicates by categorical groups was evaluated using principal component analysis (PCA) plots.

### Image quantification and statistical analysis

ImageJ (US National Institutes of Health) was used for the quantification of the fluorescence of the labeled proteins and *P. infestans* lesions. To quantify fluorescence signals from protein gels, scanned images (.gel format) were used. The intensity of the defined signal area was quantified using the Analyze -> Measure tool. The integrated density (IntDen) was used to compare intensities signal of the same dimensions (area) measurements within the same protein gel. To quantify *P. infestans* infection lesions, leaf scans were calibrated using the Analyze -> Set scale tool, using a ruler scan for calibration. All images to be compared were set to the same format (8-bit jpg) and an automatic threshold was applied (Image -> Adjust -> Threshold). Set areas applied by the threshold were measured using the Analyze -> Measure tool. The area measured in cm^2^ was used to compare lesion sizes between GST-expressing constructs and the EV control. All disease score data and fluorescence quantifications were subjected to statistical analysis using RStudio software. *P*-values were calculated using a one-way analysis of variance (one-way ANOVA). All values shown are mean values, and the error intervals represent the standard deviation (sd).

## Supplementary Material

pcad132_Supp

## Data Availability

All data generated or analyzed during this study are included in this published article and its supplementary information files. The MS proteomics data have been deposited to the ProteomeXchange Consortium via the PRIDE (Vizcaíno et al. 2016) partner repository (https://www.ebi.ac.uk/pride/archive/) with the dataset identifier PXD045884.
