# Supplementary material for: Glutathione Transferase Photoaffinity Labeling Displays GST Induction by Safeners and Pathogen Infection
Source: Plant Cell Physiol. 2023 Oct 31;65(1):128–41. doi: 10.1093/pcp/pcad132 (PMC10799724; doi:10.1093/pcp/pcad132)
Supplement: pcad132_Supp [file pcad132_supp.zip › pcp-2023-e-00235-File013.pdf]

Supplemental Tables S4 and S5 Font-Farre et al., *Glutathione transferase photoaffinity labeling displays GST induction by safeners and pathogen infection.*

Table S4 Used plasmids

Plasmid	Description	Reference
pJK268c	pL1V2-P19-F2, Binary vector.	Kourelis et al., 2020
pICH41414	pL0M-T-35S-1-41414, Level 0 Module, 35S terminator, Golden Gate plant kit	Engler et al., 2014
pICH51288	pL0M-PU-35S-TMV-3-51288, Level 0 Module, 2 x 35S promoter + TMV Ω (0.8 kb), Golden Gate plant kit	Engler et al., 2014
EC15456	pL0V-SC1-15456, Level 0 Cloning vector, Golden Gate original vector	
EC15259	pL0M-C2-6xHis-15259, Level 0 Module, His for C-terminal fusion	
EC15095	pL0M-C2-eGFP-15095, Level 0 Module, eGFP for C-terminal fusion	
pMF358	pL2M-P19-kan-2x35S::AtGSTF2-HIS	This work
pMF359	pL2M-P19-kan-2x35S::AtGSTF2-GFP	This work
pMF360	pL2M-P19-kan-2x35S::AtGSTU4-HIS	This work
pMF361	pL2M-P19-kan-2x35S::AtGSTU4-GFP	This work
pMF364	pL2M-P19-kan-2x35S::AtGSTU10-HIS	This work
pMF365	pL2M-P19-kan-2x35S::AtGSTU10-GFP	This work
pMF366	pL2M-P19-kan-2x35S::AtGSTF6-HIS	This work
pMF367	pL2M-P19-kan-2x35S::AtGSTF6-GFP	This work
pMF368	pL2M-P19-kan-2x35S::AtGSTF7-HIS	This work
pMF369	pL2M-P19-kan-2x35S::AtGSTF7-GFP	This work

Table S5 Used oligonucleotides

Name	Sequence (5’ to 3’)*
GSTU10f	**TTGAAGACAA**AATGGAGGAGAAGAAGAGCAAAG
GSTU10r	**TTGAAGACAA**CACCTGCATTTGCAGCCTGC
GSTF2f	TTGGTCTCAAATGGCAGGTATCAAAGT
GSTF2r	TTGGTCTCAGTCACCATCTTCAAAGGC
GSTF2f	TTGGTCTCATGACCTCAAGCTCTTCGAATC
RV2_GSTF2r	TTGGTCTCACACCCTGAACCTTCTCGGAAG
FW_GSTF6f	TTGGTCTCAAATGGCAGGAATCAAAG
RV_GSTF6r	TTGGTCTCACACCAAGAACCTTCTGAGCAG
FW_GSTF7f	TTGGTCTCAAATGGCAGGAATCAAAG
RV_GSTF7r	TTGGTCTCACACCAAGAACCTTCTTAGCAG
FW_GSTU8	TTGGTCTCAAATGAACCAAGAAGAGCACG
RV_GSTU8r	TTGGTCTCACACCATTAGATGTAACACTTC

*, Highlighted are the BsaI restriction sites (underlined) and the BpiI restriction sites (bold).
